# Toxin data quality: a critical examination of bacterial exotoxins and animal toxins

**DOI:** 10.1186/s13104-025-07438-2

**Published:** 2025-10-02

**Authors:** Tanja Krüger, Ivan Koludarov, Maria Littmann, Burkhard Rost, Luisa F. Jimenez-Soto

**Affiliations:** 1 Walther-Straub Institute of Pharmacology and Toxicology, Ludwig-Maximilians-Universität München, Goethestrasse 33, 80336 Munich, Germany; 2 School of Computation, Information and Technology (CIT), Department of Informatics, Unit for Bioinformatics & Computational Biology - i12, TUM (Technical University of Munich, Boltzmannstr. 3, 85748 Garching/Munich, Germany; 3 Institute for Advanced Study (TUM-IAS), Lichtenbergstr. 2a, 85748 Garching/Munich, Germany; 4 Germany & TUM School of Life Sciences Weihenstephan (WZW), Alte Akademie 8, Freising, Germany

**Keywords:** Animal toxins, Bacteria toxins, Amino acid usage, Exotoxins

## Abstract

**Objective:**

Existing toxins datasets include a mixture of proteins and toxin peptides. In this study we present two curated datasets of toxic proteins free of associated proteins: bacterial exotoxins and animal toxins. Our stringent selection criteria resulted in two data sets with only toxins that directly target or disrupt vital molecular mechanisms of their target organism. To gain insight in their properties and differences, we compared both sets of toxins to controls, and used simple biophysical features such as protein length, and amino acid composition distinguishing between evolutionary kingdoms (phyla). This approach should reveal if there is a need to consider differences present in toxin sets depending on their origin.

**Results:**

Our analysis revealed biophysical differences between animal and bacterial toxins that should not be ignored. Both toxin groups exhibited preferencial amino acid usage depending on their origin, together with higher cysteine content compared to their respective controls. The animal toxins set contains, on average, significantly shorter sequences than bacterial toxins, and had their isoelectric point shifted towards acidic pH values. We show that animal toxins and bacterial toxins possess intrinsic differences in general biophysical properties, which reinforce the necessity of segregating these datasets to ensure reliability of bioinformatics models aimed at understanding and predicting toxin characteristics.

**Supplementary Information:**

The online version contains supplementary material available at 10.1186/s13104-025-07438-2.

## Introduction

Over the past decade, numerous toxin data resources have emerged, each with a specific focus, such as animal toxins or venoms [1, 2, 3, 4]⁠, toxins from plants [5]⁠, fungi [6, 7, 8]⁠ or bacteria [9, 10, 11, 12, 13]⁠, or toxic compounds [4, 5]⁠. These resources are either highly specialized (e.g. bacterial secretion system specific) and/or contain non-toxic components (e.g. outer membrane proteins or chaperones). For several of them, their creation relied on a keyword text search (p.e. “KW_800”, in UniProt) within public sequence databases such as UniProt [14]⁠, SWISS-PROT [15]⁠, or NCBI [16]⁠.

When talking about toxins, we need to go into their definitions and classification, which can differ based on the field. For microbiologists, protein bacterial toxins are called exotoxins, and are sub-classified in *Type I* to *III* based on their location of action: extracellular (*Type I* and *II*), intracellular (*Type III*). *Type I* toxins interact with cell surface receptors, Type II disrupt cell membranes, and *Type III* enter the cell’s cytoplasm either through binding (AB toxins) or injection (effector proteins). We expand the classification to a *Type IV* toxin category responsible for degrading the extracellular matrix (ECM), essential for cellular microenvironment management [17]⁠. In the field of animal toxins, these are broadly classified into two types [18]⁠: passively delivered true poisons and actively delivered venoms. This categorization echoes the distinction between bacterial exotoxins and endotoxins, with a similar dichotomy of delivery. Our study focuses specifically on the protein components of animal venoms, drawing parallels with bacterial exotoxins.

In this resource paper, we introduce a curated dataset of bacterial exotoxins and compare them to an animal toxins set, to bridge the gap between specialized and unspecific resources. The analysis includes their nearest counterpart of non-toxic proteins: secreted proteins. By shedding light on shared traits and distinguishing features, we provide high quality data useful for development of machine learning models.

## Results and discussion

### Animal and bacterial toxins differ in similarity of sequence

The initial datasets comprised 6,810 animal and 2,417 bacterial toxins (See Table 1). Applying a sequence similarity threshold (SST) of 1.0, which eliminates identical sequences, retained about 75% of bacterial and 94% of animal proteins. More stringent reduction at SST 0.25 led to comparable numbers in both sets (1,056 bacterial, 1,125 animal), despite the initial threefold higher count of animal toxins. This indicates a higher level of diversity in the bacterial sequences than in the animal toxins sequences.

In terms of overlap, no shared toxin clusters were found between animal and bacterial proteins at SST 1.0. Even at the lowest SST (0.25), less than 1.2% of the toxin clusters included both animal and bacterial toxins.

**Table 1:** Sequence similarity clusters

		Sequence Similarity Threshold (SST)			
Number (% remaining) clusters	Raw data	1.0	0.75	0.50	0.25
All toxins (%)	9281 (100%)	8216 (89%)	4103 (44%)	2762 (30%)	2206 (24%)
Animal toxins (%)	6810 (100%)	6373 (94%)	2754 (40%)	1569 (23%)	1125 (17%)
Bacterial toxins (%)	2471 (100%)	1843 (75%)	1347 (55%)	1190 (48%)	1056 (43%)
Cluster overlap	0	0	2	3	25

Absolute number and percentage of remaining protein clusters in the animal toxins, bacterial toxins and both sets (All). In brackets, percentages compared to original unfiltered data (Raw data. Sequence similarity reduction was applied using the MMseqs2 at the thresholds of 1.00, 0.75, 0.5 and 0.25 (modus 0) [45]. The last row (Cluster overlap) shows how many clusters contain both animal and bacterial toxins

Sequence similarity at low thresholds implies but does not guarantee different structures between proteins [19]⁠. It’s likely that animal and bacterial toxins with such low similarities will differ in function.

#### Toxins use specific amino acids

**Fig. 1:**
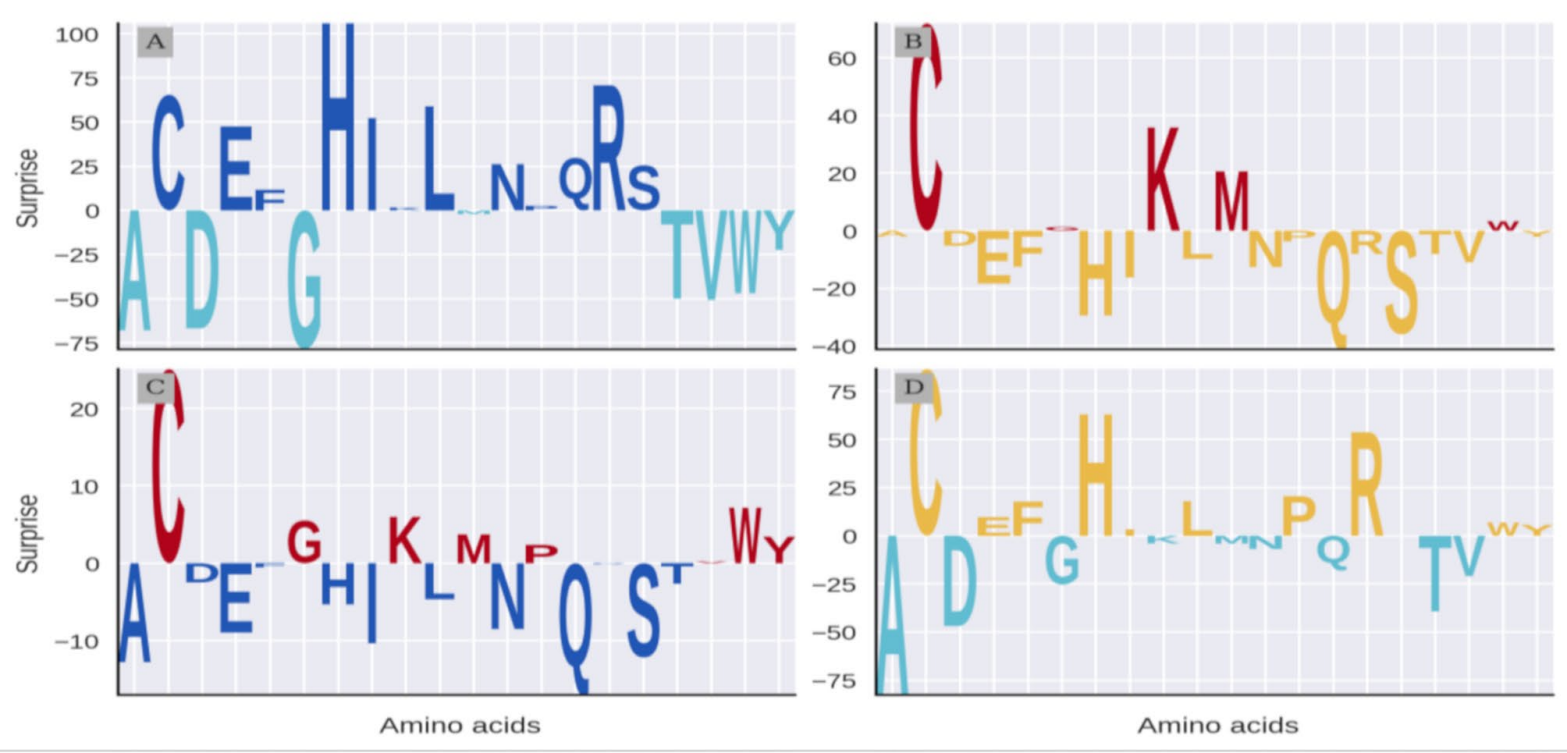
Amino acid usage varies depending on origin and function. Relative Surprise of the 20 standard amino acids occurring in (**A**) bacterial toxins (Dark blue) over secreted, non-toxins (cyan), (**B**) animal toxins (red) over secreted, non-toxins (yellow) (**C**) animal toxins (red) over bacterial toxins (dark blue) and (**D**) secreted animal non-toxins (yellow) over secreted bacterial, non-toxins (cyan). Negative values indicate a lower relative use of a amino acid relative to the full set. The plots were created using Logomaker package [48]. Scales of Surprise values allow for within-logo comparison only

We analyzed amino acid compositions using a metric termed *Surprise*, which measures the deviation of an amino acid’s (AA) composition from the mean in standard deviation units (Eq. 1). Bacterial toxins exhibited the highest *Surprise* values for histidine (H) and arginine (R) (Fig. 1A).

In contrast, the most abundant amino acids in bacterial secreted proteins (control group) were glycine (G), alanine (A), and aspartic acid (D). For animal toxins (Fig. 1B), most notable were cysteine (C) and lysine (K), whereas glutamine (Q), serine (S), and histidine (H) were more prevalent in non-toxic animal proteins, indicating an inverse trend compared to bacteria.

Direct comparison between animal and bacterial toxins (Fig. 1C) revealed cysteine (C) as most over-represented in animal toxins; with glutamine (Q) and alanine (A) being under-represented. This trend held for non-toxic secreted proteins (Fig. 1D), with cysteine over- and alanine under-represented. Cysteine stood out for all data sets. The prevalence of cysteine in animal toxins corroborates earlier findings [20]⁠. Its relevance in toxins might be related to cystein’s contribution to thermal stability [21]⁠ and protein stabilization [22]⁠. Additionally, its increased frequency in smaller proteins has been confirmed [23]⁠. Discussions of other amino acid variations are omitted for brevity.

### Toxins shorter than secreted controls

**Fig. 2:**
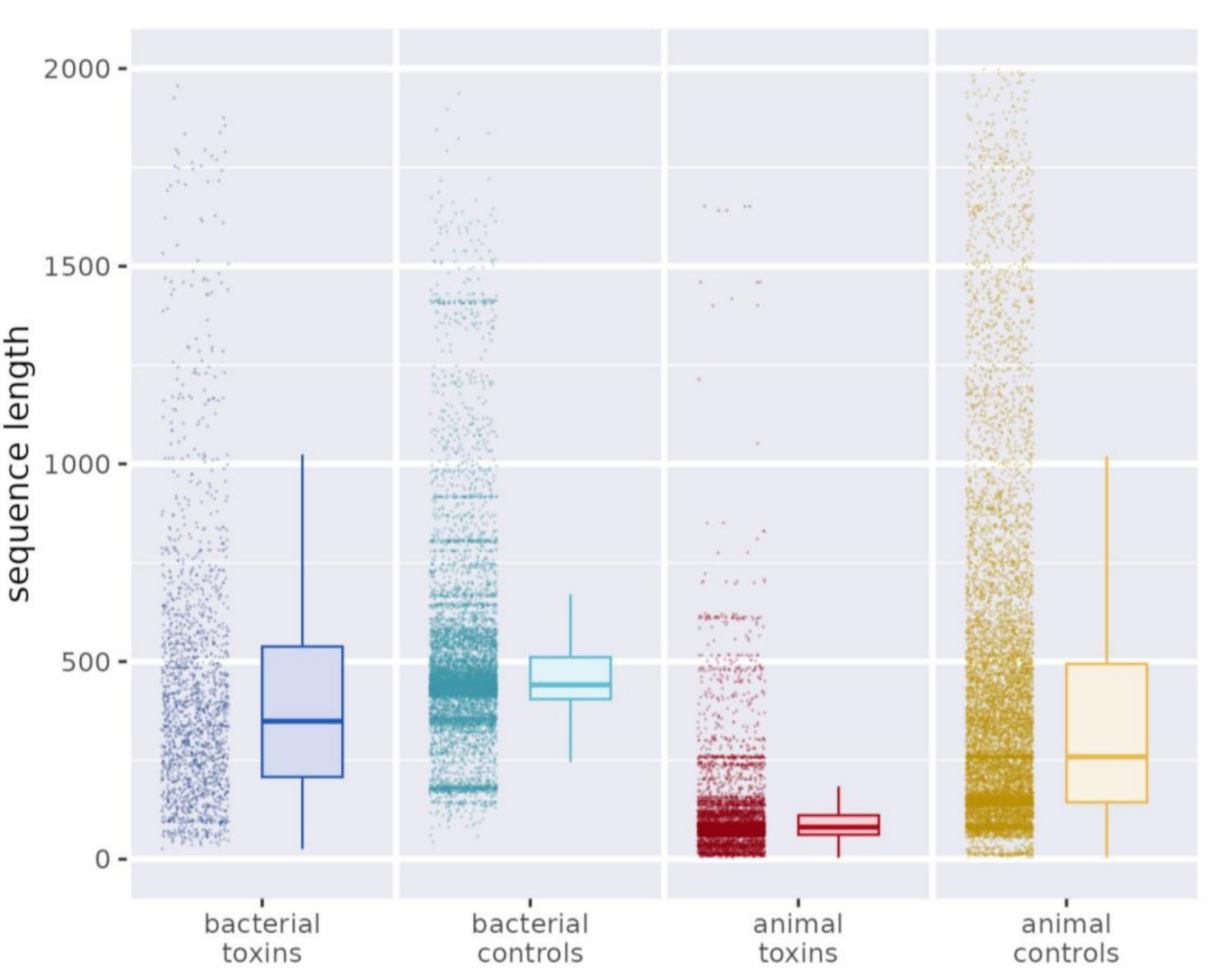
Bacterial proteins are longer than animal proteins. Sequence lengths of bacterial toxins (blue), bacterial secreted non-toxin controls (cyan), animal toxins (red), and animal secreted non-toxin controls (yellow). The y-axis is truncated at 2000 amino acids. Outliers with lengths up to 11,210 amino acids are not shown in the figure

Animal proteins in our sets were shorter than the bacterial proteins, and toxins were shorter than non-toxins (Fig. 2). Short length in animal toxins corroborates with what has been observed for sea anemones, snakes and spiders [24, 25, 26]⁠. Our datasets showing bacterial proteins longer than animal proteins is contrary to studies stating that eukaryotic proteins are longer than prokaryotic [27, 28, 29]⁠. This difference was explained by our datasets focus on secreted proteins. An expert-curated dataset employed to develop SignalP 6.0 [30]⁠⁠ confirmed our findings: secreted proteins are indeed shorter in animals than in bacteria (See Additional file 2, Fig. S2).

### Evaluation of biophysical properties: aromaticity and pI

All sets (toxins and secreted non-toxins) were similar in aromaticity; this might be related to secretion (See Additional file 3, Fig. S4). In contrast, for the isoelectric point (pI) distributions appeared bimodal with a dip around a neutral pH of 7.4 (See Additional file 3, Fig. S3), however, all toxins distributions shifted toward lower pH values compared to non-toxic proteins. This trend was stronger in bacteria (Fig. S3A) than animals (Fig. S3B), with about a third of all bacterial toxins having pI-values below 5.

Aromaticity and isoelectric points have not been thoroughly investigated for toxins. The bimodal pI distribution in other proteins with a minimum around neutral pH has been explained as a means to prevent aggregation [31, 32]⁠. Several bacterial toxins, specifically type III from AB type, are known to react to acid conditions in the endosome just before their insertion in the cytoplasm, which could explain the differences with animal toxins, which do not seem to require acid conditions for their activity. However, more research is needed to be able to define the real reason between the isoelectric point distributions shown by toxins.

To support future work in this area, we deliberately avoided subdividing the toxins by finer taxonomic or mechanistic types. Instead, our dataset contrasts animal and bacterial toxins as two overarching groups to emphasize how distinct they are in key properties. This design choice reflects our concern that mixing these biologically diverse proteins in predictive models may introduce noise and compromise model performance. We believe this perspective is essential for developers of bioinformatics tools, and we hope our resource serves as a reference point for future classifier design and validation.

## Methods

### Dataset: bacterial toxins and controls

The dataset was constructed by extracting representatives of *Type I*,* Type II*, and *Type III* toxins from UniProtKB/Swiss-Prot (RRID: SCR_021164) [15]⁠ and PubMed (RRID: SCR_004846) [16] after selecting them from literature reviews for each type of toxin. For *Type I*, we included the representatives for the Superantigen family and Enterotoxins [33]⁠.*Type II* included representatives of pore-forming toxins [34]⁠ and phospholipases [35]⁠. *Type III* toxins included AB toxins and effector proteins. For AB toxins coded in different open reading frames, the binding subunit (B) was removed. Although, it is essential for binding, localization, and delivery of the active toxin into the cytosol [36], it has no known toxicity-related enzymatic activity inside the target cell. When both subunits are encoded in one gene and secreted as one protein (e.g. Tetanus toxin), the full protein was included. Effector proteins from bacterial secretion systems were extracted from existing specialized datasets for effector proteins [37, 38, 39, 40]⁠⁠. A curation step included the removal of structural proteins, and chaperones, as they do not contain known toxicity. We have listed example proteins removed (See Additional file 1, Table S1) and kept (See Additional file 1, Table S2) from available resources, and all sources used for the revision and extraction of proteins (See Additional file 2, Table S3). We assumed that all effector proteins have a function in the manipulation of the cell metabolism. Therefore, *Type III* toxins include all known and predicted effector proteins from the secretion systems known to translocate proteins directly into the target cell, which include T3SS, T4SS, and T6SS. However, as before, we excluded structural proteins (See Additional file 1, Table S1) and proteins involved in the translocation. To get a better idea of the criteria used, see Table S1 and S2 in the Additional File 1.

Based on our operational definition of toxins, we have expanded the classification of bacterial toxins to a *Type IV*, which included bacterial proteins degrading extracellular matrix (ECM). By degrading the ECM they have a potential effect on cell behavior. Representatives of *Type IV* toxins included enzymes with direct effect on ECM proteins such as collagenases, siacylases, and metalloproteases.

For bacterial secreted proteins (control) with no known toxin activity, we selected all secreted proteins, non-membrane associated, and no outer membrane vesicles (OMV) associated from monoderms and diderms, available in the PSORTb 3.0 database (accessed June 2021) [41]⁠. This database was chosen because its classification considers the potential membrane association of secreted proteins, which other databases do not. This allows us to select the most similar proteins to bacterial toxins. We removed all proteins that contained “Fragment” within the identification.

### Dataset: animal toxins and controls

We based the dataset of animal venom toxins on the expert-curated Tox-Prot [42]⁠, itself is a subset of UniProt (accessed on Dec 2022). We included venom proteins from jellyfish, snails, centipedes, insects, arachnids, fish, mammals and snakes. We added proteins retrieved from our own studies on three-finger toxins (3FTs) in snakes [43]⁠ and of our own recent annotation of hymenopteran venoms [44]⁠.

For the control set of secreted, animal proteins, we selected all secreted [KW-0964], non-toxic proteins [NOT KW-0800] from the same organism as in the animal venoms, based on their *Organism ID* as defined in UniProt [14]⁠ (accessed July 2023). For animals, we chose UniProt as better suited because of the better prediction of subcellular localization for eukaryotic proteins. We removed all proteins that contained “Fragment” within the identification.

### Dataset comparison

To address a possible bias from sequence redundancy, we excluded identical sequences within each of the sets through the alignment method *MMseqs2* [45]⁠. We applied the default parameter setting from the easy-cluster option at a sequence similarity threshold of 1.00, and alignment coverage modus of 0 [45]⁠. At modus 0, the alignments cover both the query- and the target sequence at the selected coverage threshold. We kept only the cluster-representatives for each cluster.

To investigate the remaining sequence diversity within the sets, we used MMseqs2 clustered at three different thresholds of 0.75, 0.50, and 0.25. Subsequently we calculated the number of clusters (n)that were similar given a particular threshold (thresh) as follows:

1 $$\eqalign{{n}_{\text{thresh}}& \text{=n}{\left(\text{animaltoxins}\right)}_{\text{thresh}}\text{+n}{\left(\text{bacterialtoxins}\right)}_{\text{thresh}}\cr & -n{\left(\text{alltoxins}\right)}_{\text{thresh}}}$$

For each dataset, we calculated the amino acid composition (percentage of any of the 20 native amino acids), average sequence length (number of residues per protein), the aromaticity (percentage of phenylalanine, tryptophan and tyrosine) and the isoelectric points (pI) after Bjellqvist [46]⁠. The pI, amino acid composition, average sequence length and aromaticity were calculated after removal of duplicates from the raw data using Biopython (RRID: SCR_007173).

To visualize the differences in amino acid composition between datasets, we calculated the *Surprise* (Eq. 1) per amino acid, defined as follows:

2 $$\text{Surprise}\left(\text{AA}\right) =\frac{{\mu\:}_{\text{dataset}}-{\mu\:}_{\text{mergeddatasets}}}{{\sigma\:}_{\text{mergeddatasets}}}$$

with AA as one of the 20 amino acids; µ_data1_, as the average percentage of AA in data set 1, and µ_mergeddatasets_ as the average for data set 1 minus background (both sets combined); while σ_mergeddatasets_ described the standard deviation.

We first computed the amino acid composition in the single and merged datasets. Then, we generated a normal distribution by bootstrapping with 10,000 samples to calculate the mean and standard deviations. Finally, the *Surprise* values determined the height of each amino acid letter (one-letter code) using the python package Logomaker [47]⁠⁠ .

### Limitations

The following limitations need to be considered: Our bacterial datasets are biased as a result of databases enriched in bacteria that can be cultured in the laboratory and their relevance as human pathogens. Future analyses addressing the biases in datasets available, like metagenomic data, or computational predictions for identification of toxins from unculturable organisms, will be valuable approaches. What our data cannot tell us is if these differences will also be valid for toxins from other bacteria, fungi, or plants. While we acknowledge reader interest in fungal toxin comparisons, our research group lacks expertise in mycotoxins. However, we have conducted a preliminary analysis comparing 288 unique fungal peptide toxins with the animal and bacterial toxins studied here. The methods and results of this analysis are included in the supplementary files (Additional file 4, Figures S5 to S8). Given the limited size of the fungal dataset, we decided not to include these findings in the main results section.

## Supplementary Information

Below is the link to the electronic supplementary material.

Supplementary Material 1

Supplementary Material 2

Supplementary Material 3

Supplementary Material 4

## Data Availability

The datasets generated and analysed during the current study are available in the LMU repository Open Data LMU (RRID: SCR_025005) under the DOI: 10.5282/ubm/data.423. For the supplementary data containing the fungi peptide toxins dataset, code for analysis and supplementary file, you can find them under DOI: https://data.ub.uni-muenchen.de/555/. If updated, the repository will allow access to the updated and previous version.

## Abbreviations

AA: Amino acids
A: Alanine
R: Arginine
N: Asparagine
D: Aspartic acid
C: Cysteine
E: Glutamic acid
Q: Glutamine
G: Glycine
H: Histidine
I: Isoleucine
K: Lysine
L: Leucine
M: Methionine
F: Phenylalanine
P: Proline
S: Serine
T: Threonine
W: Tryptophan
V: Valine
ECM: Extracellular matrix
IQR: Inter quartile range
OMV: Outer Membrane Vesicles
pI: Isoelectric point
pKa: Acid dissociation constant
T3SS: Type 3 secretion system
T4SS: Type 4 secretion system
T6SS: Type 6 secretion system
SST: sequence similarity threshold
