# Supplementary material for: Toxin data quality: a critical examination of bacterial exotoxins and animal toxins
Source: BMC Res Notes. 2025 Oct 2;18:418. doi: 10.1186/s13104-025-07438-2 (PMC12492933; doi:10.1186/s13104-025-07438-2)
Supplement: Supplementary file 1 — Supplementary Material 1 [file 13104_2025_7438_MOESM1_ESM.pdf]

Examples of toxin curation

Table S1: Example proteins removed because they do not fulfill our definition of toxins

Protein(s) removed	Reason	Reference
VirB7, VirB9, VirB10 homologues of T4SS	Structural protein T4SS	[1]
CagF (*H. pylori*)	Chaperone of CagA toxin	[2]
BipD (*Burkholderia spp*)	Tip protein of T3SS	[3]
BslA (*Burkholderia spp*)	Needle protein of T3SS	[3]
Por*, Gld* and Spr* (*Porphyromonas* spp)	Structural proteins T9SS	[4]
Tss*	Structural proteins T6SS	[5]
EsaA, EssA, EssB, EssC, EsaB	Structural proteins T7SS	[6]

Table S2: Example proteins kept because they fulfill our definition of toxins

Protein(s) kept	Reason	Reference
PtxA	Active toxin injected in target cells	[7]
PtxB – PtxE from T4SS *Bordetella pertussis*	B subunit of pertussis toxin able to trigger cellular processes similar to Type I toxins	[7], [8]
Mayor and Minor translocon proteins (p.e. BipB, BipC)	Part of the translocon of the T3SS. They disrupt the membrane of cells, sharing characteristics with pore forming toxins.	[3]
VgrG	T6SS secreted protein with effect activity on target cells	[9]
T-DNA border endonuclease VirD2	Translocated into plant cells with DNA	[10]
Tir	Inserts in the membrane and acts as receptor , manipulates calcium influx	[11]
Tse* proteins	Effector toxins from T6SS	[5]
EsxA (*Streptococcus spp)*	Effector protein from T7SS	[6]

Bibliography (Table S1 and S2)

[1] K. Wallden, A. Rivera-Calzada, and G. Waksman, “Type IV secretion systems: versatility and diversity in function,” *Cell. Microbiol.*, vol. 12, no. 9, p. 1203, Sep. 2010, doi: 10.1111/J.1462-5822.2010.01499.X.

[2] I. Pattis, E. Weiss, R. Laugks, R. Haas, and W. Fischer, “The Helicobacter pylori CagF protein is a type IV secretion chaperone-like molecule that binds close to the C-terminal secretion signal of the CagA effector protein,” *Microbiology*, vol. 153, no. Pt 9, pp. 2896–2909, 2007.

[3] P. J. Matteï, E. Faudry, V. Job, T. Izoré, I. Attree, and A. Dessen, “Membrane targeting and pore formation by the type III secretion system translocon,” *FEBS Journal*, vol. 278, no. 3. John Wiley & Sons, Ltd, pp. 414–426, Feb. 01, 2011, doi: 10.1111/j.1742-4658.2010.07974.x.

[4] M. Paillat, I. L. Silva, E. Cascales, and T. Doan, “A journey with type IX secretion system effectors: selection, transport, processing and activities,” *Microbiol. (United Kingdom)*, vol. 169, no. 4, p. 001320, Apr. 2023, doi: 10.1099/MIC.0.001320/CITE/REFWORKS.

[5] K. W. Yu, P. Xue, Y. Fu, and L. Yang, “T6SS Mediated Stress Responses for Bacterial Environmental Survival and Host Adaptation,” *Int. J. Mol. Sci. 2021, Vol. 22, Page 478*, vol. 22, no. 2, p. 478, Jan. 2021, doi: 10.3390/IJMS22020478.

[6] B. L. Spencer, U. Tak, J. C. Mendonça, P. E. Nagao, M. Niederweis, and K. S. Doran, “A type VII secretion system in Group B Streptococcus mediates cytotoxicity and virulence,” *PLoS Pathog.*, vol. 17, no. 12, Dec. 2021, doi: 10.1371/JOURNAL.PPAT.1010121.

[7] S. Mangmool and H. Kurose, “Gi/o Protein-Dependent and -Independent Actions of Pertussis Toxin (PTX),” *Toxins (Basel).*, vol. 3, no. 7, p. 884, Jul. 2011, doi: 10.3390/TOXINS3070884.

[8] M. N. M. Santos, S. T. Cho, C. F. Wu, C. J. Chang, C. H. Kuo, and E. M. Lai, “Redundancy and Specificity of Type VI Secretion vgrG Loci in Antibacterial Activity of Agrobacterium tumefaciens 1D1609 Strain,” *Front. Microbiol.*, vol. 10, p. 497665, Jan. 2020, doi: 10.3389/FMICB.2019.03004/BIBTEX.

[9] J. Lennings, T. E. West, and S. Schwarz, “The Burkholderia Type VI secretion system 5: Composition, regulation and role in virulence,” *Front. Microbiol.*, vol. 10, no. JAN, p. 3339, 2019, doi: 10.3389/FMICB.2018.03339/BIBTEX.

[10] M. Van Kregten, B. I. Lindhout, P. J. J. Hooykaas, and B. J. Van Der Zaal, “Agrobacterium-Mediated T-DNA Transfer and Integration by Minimal VirD2 Consisting of the Relaxase Domain and a Type IV Secretion System Translocation Signal,” *Mol. Plant-Microbe Interact. MPMI*, vol. 22, no. 11, pp. 1356–1365, 2009, doi: 10.1094/MPMI.

[11] L. Krampen *et al.*, “Revealing the mechanisms of membrane protein export by virulence-associated bacterial secretion systems,” *Nat. Commun. 2018 91*, vol. 9, no. 1, pp. 1–10, Aug. 2018, doi: 10.1038/s41467-018-05969-w.
