# Supplementary material for: Toxin data quality: a critical examination of bacterial exotoxins and animal toxins
Source: BMC Res Notes. 2025 Oct 2;18:418. doi: 10.1186/s13104-025-07438-2 (PMC12492933; doi:10.1186/s13104-025-07438-2)
Supplement: Supplementary file 2 — Supplementary Material 2 [file 13104_2025_7438_MOESM2_ESM.pdf]

Publications of bacterial toxins

Table S3: **Publications consulted for the creation of the bacterial toxin dataset.**

Type 1 Toxins	[1]–[3]
Type 2 Toxins	[4]–[12] and http://www.tcdb.org/superfamily.php (Transporter Classification Database (RRID:SCR_004490))
Type 3 Toxins	[13], [14], [23], [15]–[22]
Type 4 Toxins	[24]–[29]
Databases/ Datasets	[14], [30]–[42]

Not all databases or links in the publications were accessible. Several links to the datasets were broken. Other resources are in Table S1 and Table S2

Bibliography

[1] N. Stich, M. Waclavicek, N. Model, and M. M. Eibl, "Staphylococcal superantigen (TSST-1) mutant analysis reveals that t cell activation is required for biological effects in the rabbit including the cytokine storm," *Toxins (Basel).*, vol. 2, no. 9, pp. 2272–2288, Sep. 2010.

[2] B. A. Shannon, J. K. MCCormick, and P. M. Schlievert, "Toxins and Superantigens of Group A Streptococci," *Microbiol. Spectr.*, vol. 7, no. 1, Jan. 2019.

[3] S. Sriskandan, L. Faulkner, and P. Hopkins, "Streptococcus pyogenes: Insight into the function of the streptococcal superantigens," *Int. J. Biochem. Cell Biol.*, vol. 39, no. 1, pp. 12–19, 2007.

[4] P. Stanley, V. Koronakis, and C. Hughes, " Acylation of Escherichia coli Hemolysin: A Unique Protein Lipidation Mechanism Underlying Toxin Function ," *Microbiol. Mol. Biol. Rev.*, vol. 62, no. 2, pp. 309–333, Jun. 1998.

[5] M. W. Parker and S. C. Feil, "Pore-forming protein toxins: from structure to function," *Prog. Biophys. Mol. Biol.*, vol. 88, no. 1, pp. 91–142, May 2005.

[6] Y. Li *et al.*, "Structural Basis of the Pore-Forming Toxin/Membrane Interaction," *Toxins (Basel).*, vol. 13, no. 2, Feb. 2021.

[7] B. Geny and M. R. Popoff, "Bacterial protein toxins and lipids: pore formation or toxin entry into cells," *Biol. Cell*, vol. 98, no. 11, 2006.

[8] M. Pleckaityte, "Cholesterol-Dependent Cytolysins Produced by Vaginal Bacteria: Certainties and Controversies," *Front. Cell. Infect. Microbiol.*, vol. 9, Jan. 2020.

[9] M. Flores-Díaz, L. Monturiol-Gross, C. Naylor, A. Alape-Girón, and A. Flieger, "Bacterial Sphingomyelinases and Phospholipases as Virulence Factors," *Microbiol. Mol. Biol. Rev.*, vol. 80, no. 3, pp. 597–628, Sep. 2016.

[10] R. W. Titball, "Bacterial phospholipases C," *Microbiological Reviews*, vol. 57, no. 2. pp. 347–366, 1993.

[11] C. J. Rosado *et al.*, "The MACPF/CDC family of pore-forming toxins," *Cell. Microbiol.*, vol. 10, no. 9, pp. 1765–1774, 2008.

[12] M. Manich *et al.*, "Clostridium perfringens Delta Toxin Is Sequence Related to Beta Toxin, NetB, and Staphylococcus Pore-Forming Toxins, but Shows Functional Differences," *PLoS One*, vol. 3, no. 11, p. e3764, Nov. 2008.

[13] R. Samudrala, F. Heffron, and J. E. McDermott, "Accurate prediction of secreted substrates and identification of a conserved putative secretion signal for type iii secretion systems," *PLoS Pathog.*, vol. 5, no. 4, p. e1000375, Apr. 2009.

[14] X. Dong, X. Lu, and Z. Zhang, "BEAN 2.0: An integrated web resource for the identification and functional analysis of type III secreted effectors," *Database*, vol. 2015, p. bav064, Jun. 2015.

[15] S. Lockwood *et al.*, "Identification of Anaplasma marginale type IV secretion system effector proteins," *PLoS One*, vol. 6, no. 11, 2011.

[16] S. S. Abby, J. Cury, J. Guglielmini, B. Néron, M. Touchon, and E. P. C. Rocha, "Identification of protein secretion systems in bacterial genomes," *Sci. Reports 2016 61*, vol. 6, no. 1, pp. 1–14, Mar. 2016.

[17] J. Monjarás Feria and M. A. Valvano, "An Overview of Anti-Eukaryotic T6SS Effectors," *Front. Cell. Infect. Microbiol.*, vol. 10, Oct. 2020.

[18] Z. E. Ashari, K. A. Brayton, and S. L. Broschat, "Prediction of T4SS effector proteins for anaplasma phagocytophilum using OPT4e, A new software tool," *Front. Microbiol.*, vol. 10, no. JUN, 2019.

[19] G. N. Schroeder, "The toolbox for uncovering the functions of legionella Dot/Icm Type IVb secretion system effectors: Current state and future directions," *Frontiers in Cellular and Infection Microbiology*, vol. 7, no. JAN. 2018.

[20] R. Shrivastava and J. F. Miller, "Virulence Factor Secretion by Bordetella Species," *Curr. Opin. Microbiol.*, vol. 12, no. 1, p. 88, Feb. 2009.

[21] A. Zalguizuri, G. Caetano-Anollés, and V. C. Lepek, "Phylogenetic profiling, an untapped resource for the prediction of secreted proteins and its complementation with sequence-based classifiers in bacterial type III, IV and VI secretion systems," *Brief. Bioinform.*, vol. 20, no. 4, pp. 1395–1402, Mar. 2018.

[22] L. Terradot and G. Waksman, "Architecture of the Helicobacter pylori Cag-type IV secretion system," *FEBS J*, vol. 278, no. 8, pp. 1213–1222, 2011.

[23] B. T. Ho, Y. Fu, T. G. Dong, and J. J. Mekalanos, “Vibrio cholerae type 6 secretion system effector trafficking in target bacterial cells,” *Proc. Natl. Acad. Sci. U. S. A.*, vol. 114, no. 35, pp. 9427–9432, Aug. 2017.

[24] H. Kavermann *et al.*, “Identification and characterization of Helicobacter pylori genes essential for gastric colonization,” *J.Exp.Med.*, vol. 197, no. 7, pp. 813–822, 2003.

[25] C. R. Rasmussen-Ivey, M. J. Figueras, D. McGarey, and M. R. Liles, “Virulence factors of Aeromonas hydrophila: In the wake of reclassification,” *Front. Microbiol.*, vol. 7, no. AUG, p. 1337, Aug. 2016.

[26] A. M. Lasica, M. Ksiazek, M. Madej, and J. Potempa, “The Type IX Secretion System (T9SS): Highlights and Recent Insights into Its Structure and Function,” *Frontiers in Cellular and Infection Microbiology*, vol. 7, no. MAY. 2017.

[27] M. Paillat, I. L. Silva, E. Cascales, and T. Doan, “A journey with type IX secretion system effectors: selection, transport, processing and activities,” *Microbiol. (United Kingdom)*, vol. 169, no. 4, p. 001320, Apr. 2023.

[28] J. K. Rudkin, R. M. McLoughlin, A. Preston, and R. C. Massey, “Bacterial toxins: Offensive, defensive, or something else altogether?,” *PLoS Pathogens*, vol. 13, no. 9. Public Library of Science, p. e1006452, 01-Sep-2017.

[29] Y. T. Lin, C. T. Wang, and B. L. Chiang, “Role of bacterial pathogens in atopic dermatitis,” *Clinical Reviews in Allergy and Immunology*, vol. 33, no. 3. 2007.

[30] S. S. Negi *et al.*, “Functional classification of protein toxins as a basis for bioinformatic screening,” *Sci. Rep.*, vol. 7, no. 1, p. 13940, Dec. 2017.

[31] C. E. Zhou, J. Smith, M. Lam, A. Zemla, M. D. Dyer, and T. Slezak, “MvirDB - A microbial database of protein toxins, virulence factors and antibiotic resistance genes for bio-defence applications,” *Nucleic Acids Res.*, vol. 35, no. SUPPL. 1, 2007.

[32] D. Bi, L. Liu, C. Tai, Z. Deng, K. Rajakumar, and H. Y. Ou, “SecReT4: a web-based bacterial type IV secretion system resource,” *Nucleic Acids Res.*, vol. 41, no. D1, pp. D660–D665, Jan. 2013.

[33] R. C. Souza *et al.*, “AtlasT4SS: A curated database for type IV secretion systems,” *BMC Microbiol.*, vol. 12, no. 1, pp. 1–11, Aug. 2012.

[34] V. Eichinger, T. Nussbaumer, A. Platzer, M. A. Jehl, R. Arnold, and T. Rattei, “EffectiveDB - Updates and novel features for a better annotation of bacterial secreted proteins and Type III, IV, VI secretion systems,” *Nucleic Acids Res.*, vol. 44, no. D1, 2016.

[35] X. Pan *et al.*, “ToxDL: deep learning using primary structure and domain embeddings for assessing protein toxicity,” *Bioinformatics*, vol. 36, no. 21, pp. 5159–5168, Jan. 2021.

[36] F. Jungo and A. Bairoch, “Tox-Prot, the toxin protein annotation program of the Swiss-Prot protein knowledgebase,” *Toxicon*, vol. 45, no. 3, pp. 293–301, 2005.

[37] A. Chakraborty, S. Ghosh, G. Chowdhary, U. Maulik, and S. Chakrabarti, “DBETH: A database of bacterial exotoxins for human,” *Nucleic Acids Res.*, vol. 40, no. D1, 2012.

[38] S. Muthiah, D. Datta, M. R. Islam, P. Butler, A. Warren, and N. Ramakrishnan, “ProtTox: Toxin identification from protein sequences,” *bioRxiv*, 2020.

[39] E. Lim *et al.*, “T3DB: A comprehensively annotated database of common toxins and their targets,” *Nucleic Acids Res.*, vol. 38, no. SUPPL.1, 2009.

[40] L. de Nies *et al.*, “PathoFact: a pipeline for the prediction of virulence factors and antimicrobial resistance genes in metagenomic data,” *Microbiome 2021 91*, vol. 9, no. 1, pp. 1–14, Feb. 2021.

[41] L. C. B. Barbosa, S. S. Garrido, and R. Marchetto, “BtoxDB: A comprehensive database of protein structural data on toxin-antitoxin systems,” *Comput. Biol. Med.*, vol. 58, 2015.

[42] B. Liu, D. Zheng, Q. Jin, L. Chen, and J. Yang, “VFDB 2019: A comparative pathogenomic platform with an interactive web interface,” *Nucleic Acids Res.*, vol. 47, no. D1, 2019.
