# Supplementary material for: Toxin data quality: a critical examination of bacterial exotoxins and animal toxins
Source: BMC Res Notes. 2025 Oct 2;18:418. doi: 10.1186/s13104-025-07438-2 (PMC12492933; doi:10.1186/s13104-025-07438-2)
Supplement: Supplementary file 3 — Supplementary Material 3 [file 13104_2025_7438_MOESM3_ESM.pdf]

Toxin Data Quality: A Critical Examination of Bacterial Exotoxins and Animal Toxins

Supplementary analysis of datasets

Description:

Figure S1: More extensive length analysis including density plots.
Figure S2: Comparative length analysis of an established dataset that includes both secreted and non-secreted proteins.
Figure S3: Analysis of protein isoelectric points in toxins and control proteins.
Figure S4: Analysis of protein aromaticity in toxins and control proteins.

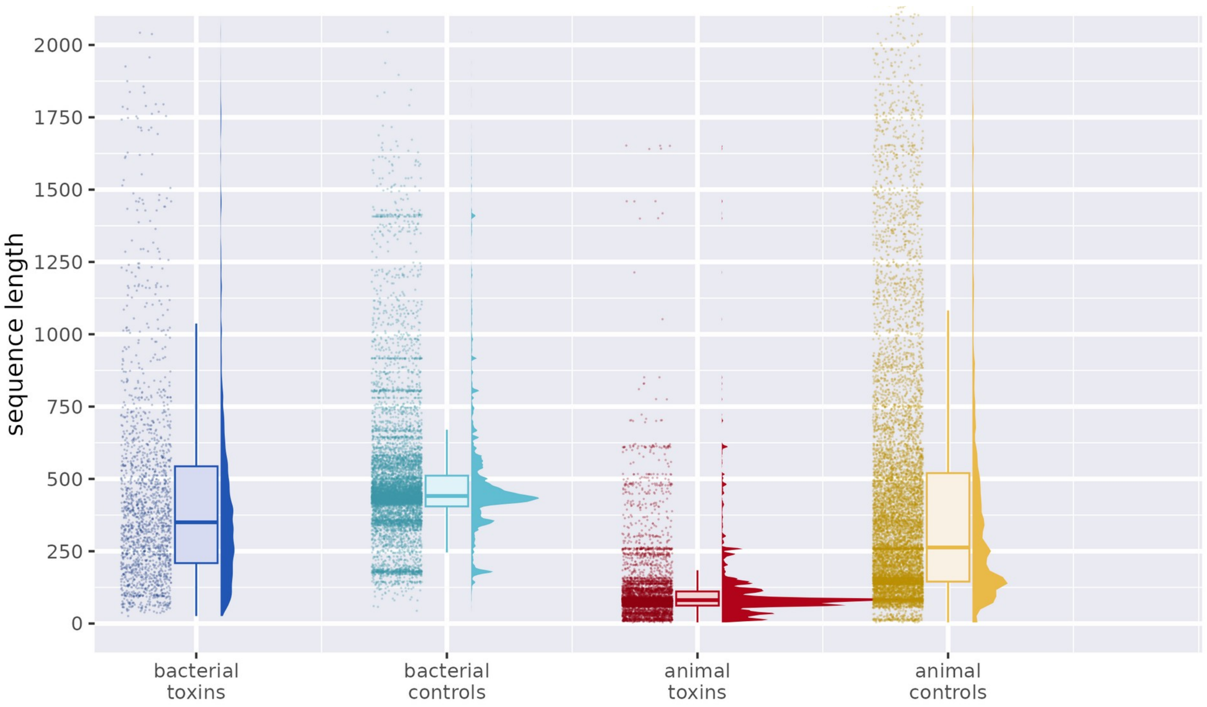

Fig S1: Bacterial proteins are longer than animal proteins.

Sequence lengths of four sets of proteins: bacterial toxins (blue), bacterial secreted non-toxin controls (cyan), animal toxins (red), and animal secreted non-toxin controls (yellow). Three representations of the values are shown: a scatter plot, a boxplot, and a histogram. Each point in the scatter plot represents a protein. The boxplot shows the median and the interquartile range (IQR) of each set. The histogram shows the normalized frequency of lengths for each set. The y-axis is truncated at 2000 amino acids. Outliers with lengths up to 11210 amino acids are not shown.

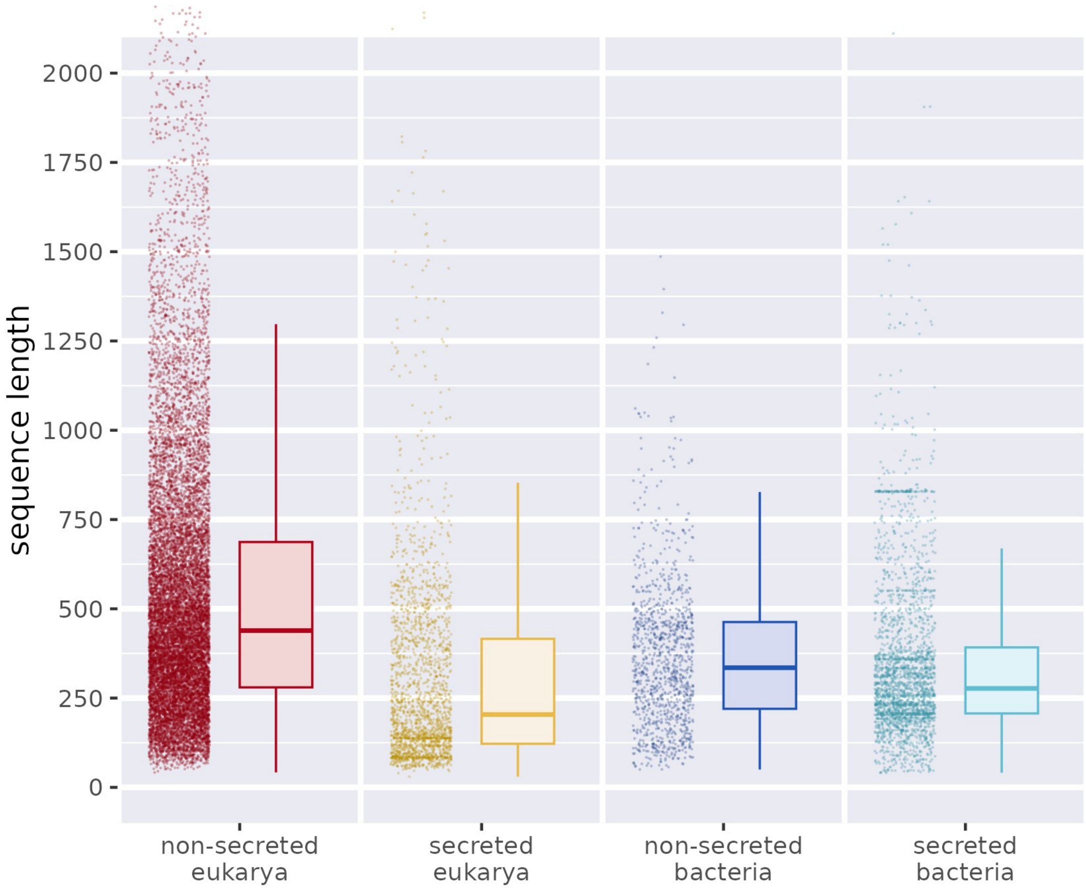

Fig. S2: SignalP training set shows similar length distribution of secreted proteins. Sequence lengths of SignalP 6.0 (cite) training data separated into four sets of proteins: non-secreted eukarya (red), secreted eukarya (yellow) non-secreted bacteria (blue), secreted bacteria (cyan). A scatterplot and a boxplot are presented. Each point in the scatter plot represents a protein. The boxplot shows the median and the interquartile range (IQR) of each set. The y-axis is truncated at 2000 amino acids.

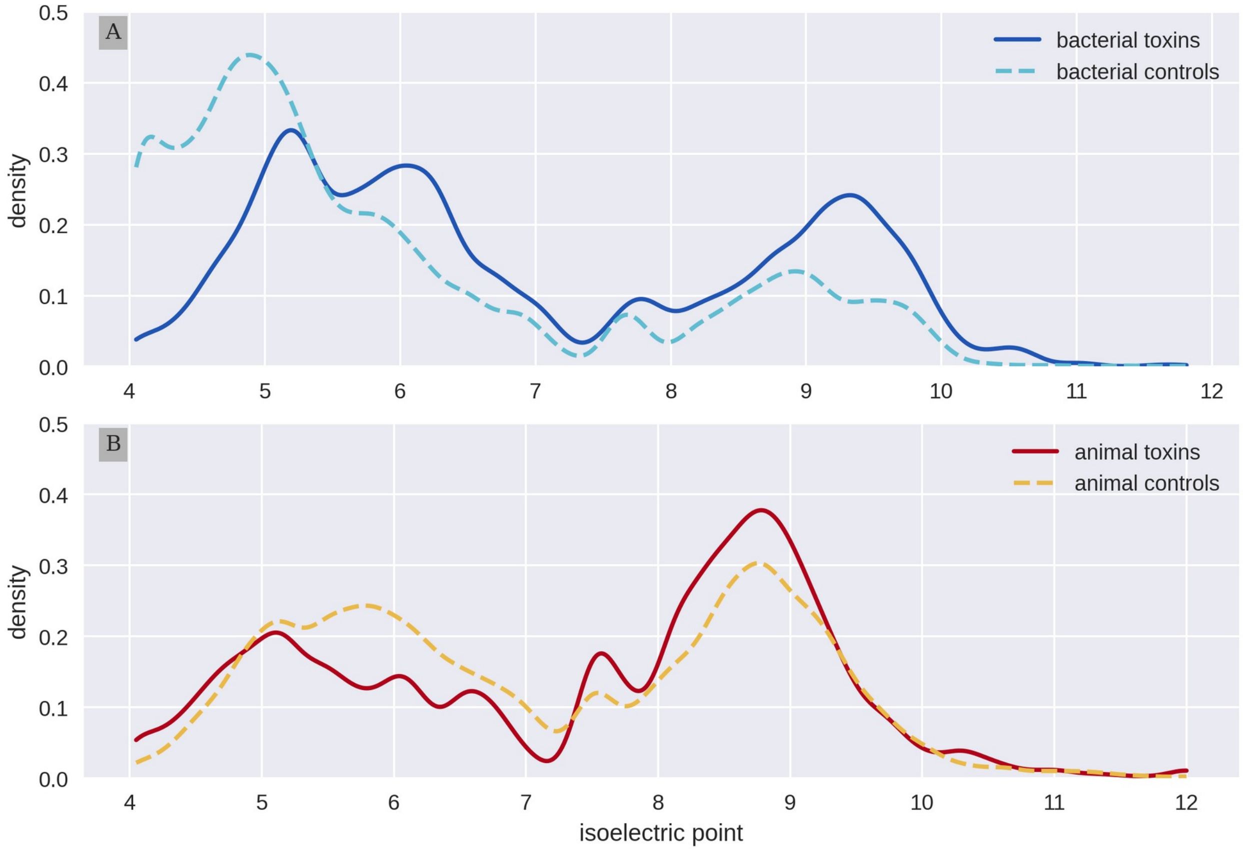

Fig. S3: Isoelectric points shift in bacterial secreted proteins

Pairwise comparison of the average isoelectric points of proteins, calculation after Bjellqvist 1993.(A) bacterial toxins compared to the control (bacterial, secreted non-toxins),(B) animal toxins compared to their control set (animal, secreted, non-toxins).

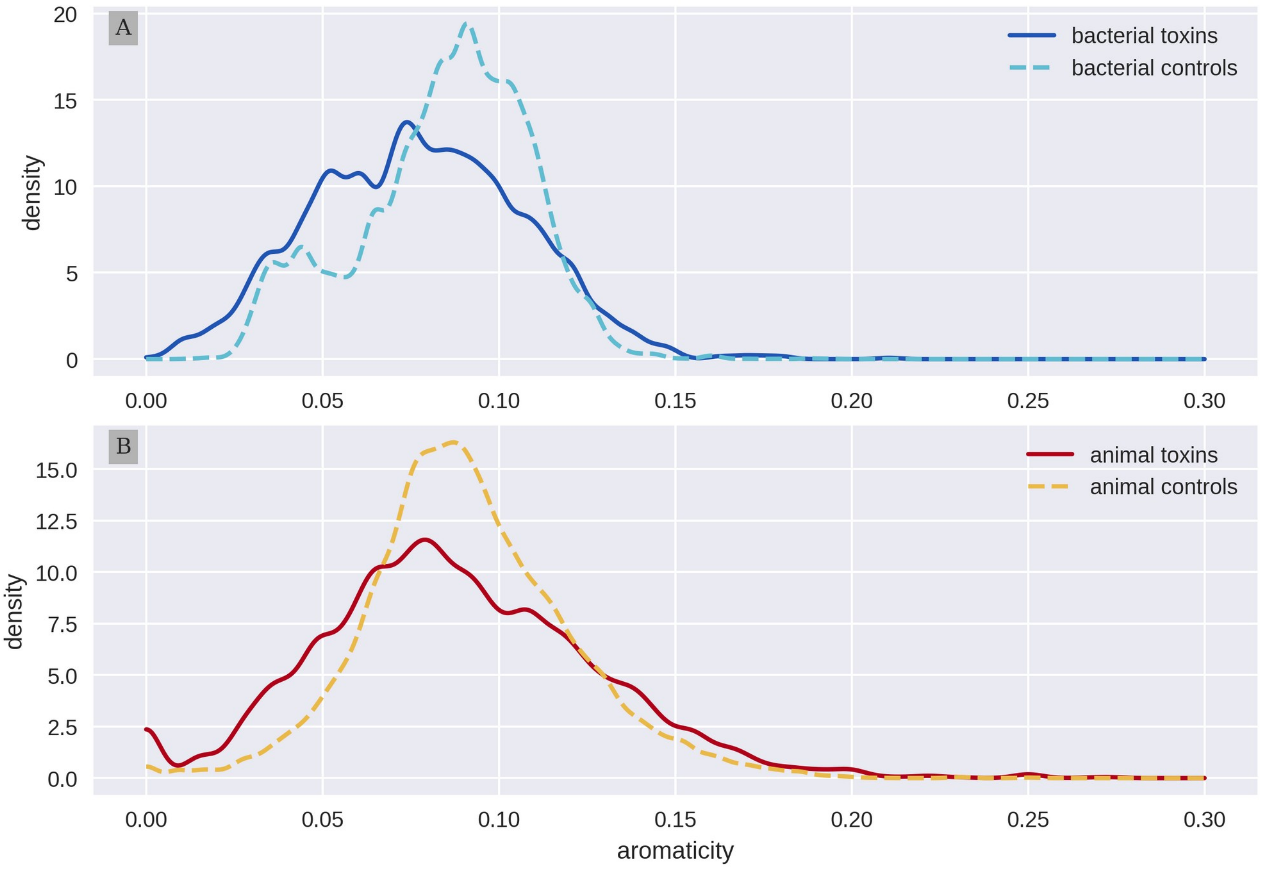

Fig. S4: All toxins reveal similar aromaticity. Pairwise comparison of the average aromaticity of proteins. Here aromaticity is the relative frequency of phenylalanine, tryptophan and tyrosine. A) bacterial toxins compared to their control (bacterial, secreted non-toxins),(B) animal toxins compared to their control set (animal, secreted, non-toxins)
