# Supplementary material for: Toxin data quality: a critical examination of bacterial exotoxins and animal toxins
Source: BMC Res Notes. 2025 Oct 2;18:418. doi: 10.1186/s13104-025-07438-2 (PMC12492933; doi:10.1186/s13104-025-07438-2)
Supplement: Supplementary file 4 — Supplementary Material 4 [file 13104_2025_7438_MOESM4_ESM.pdf]

Supplementary analysis of fungal protein toxins

Description:

Figure S5: Length analysis of fungal toxins.
Figure S6: Analysis of amino acid composition of fungal toxins.
Figure S7: Analysis of protein isoelectric points in fungal toxins.
Figure S8: Analysis of protein aromaticity in fungal toxins.

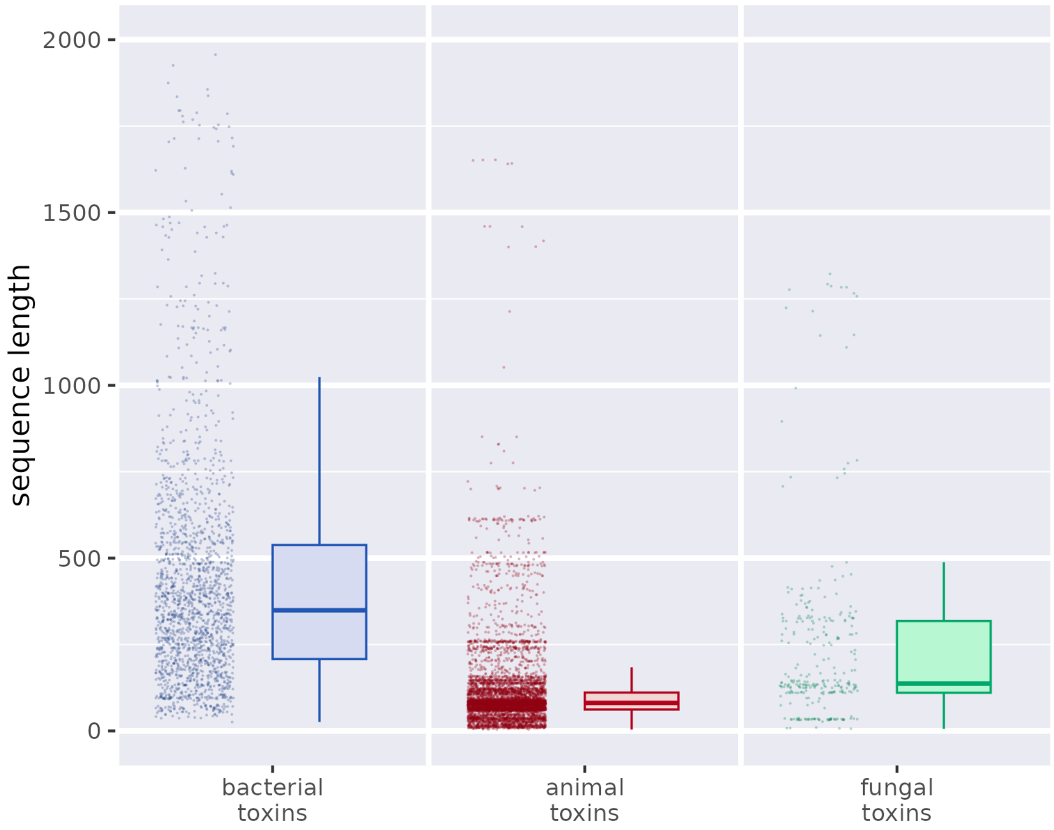

Fig. S5: Fungal toxins differ in length.

Sequence lengths of fungal toxins (green) bacterial toxins (blue), and animal toxins (red). The y-axis is truncated at 2000 amino acids. Outliers with lengths up to 11210 amino acids are not shown in the figure.

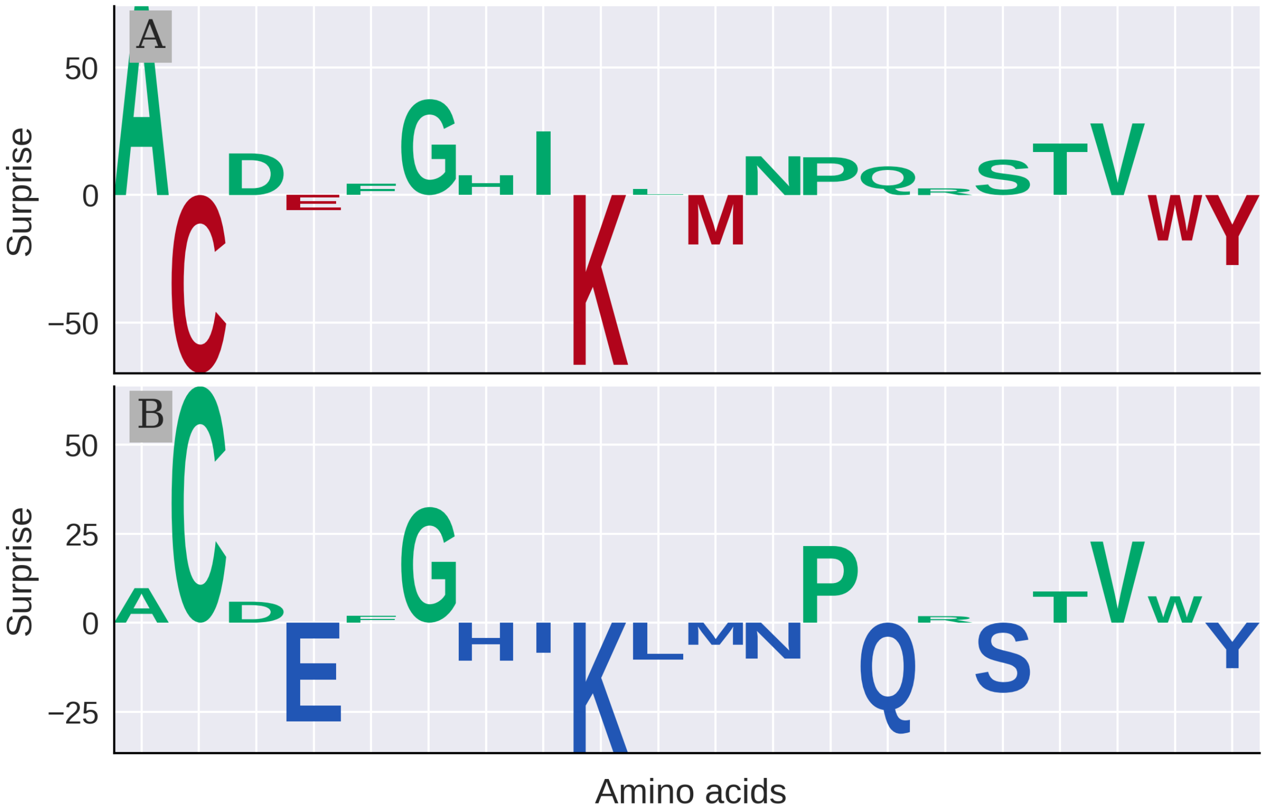

Fig. S6: Amino acid usage of fungi distinct from other toxin origins.

Relative Surprise of the 20 standard amino acids occurring in (A) fungi toxins (green) over animal toxins (red), (B) fungal toxins (green) over bacterial toxins (dark blue). Negative values indicate a lower relative use of a amino acid relative to the full set. The plots were created using Logomaker package. Scales of Surprise values allow for within-logo comparison only.

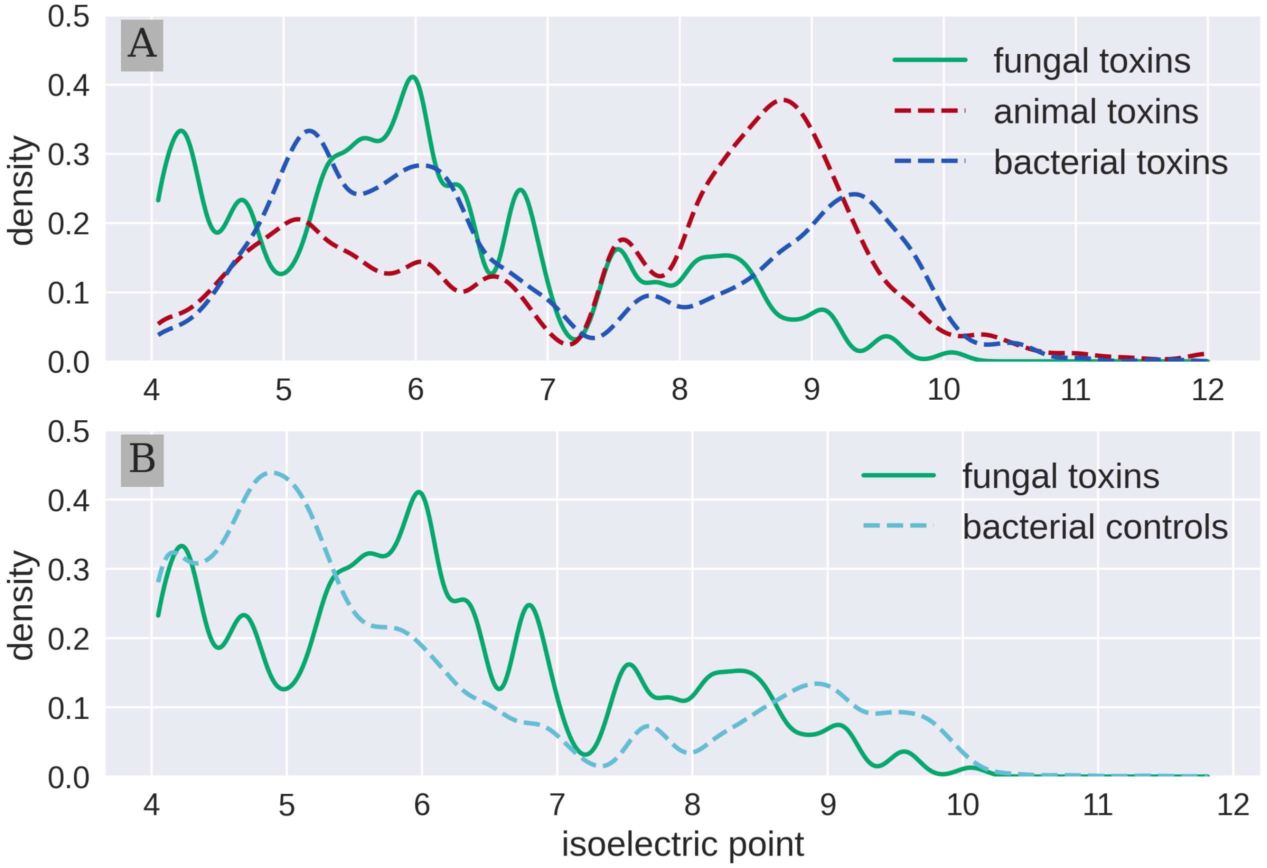

Fig. S7: Isoelectric points shift in fungal toxins

Comparison of the average isoelectric points of proteins, calculation after Bjellqvist 1993. (A) Fungal toxins compared to animal and bacterial toxins, (B) Fungal toxins compared to bacterial control set.

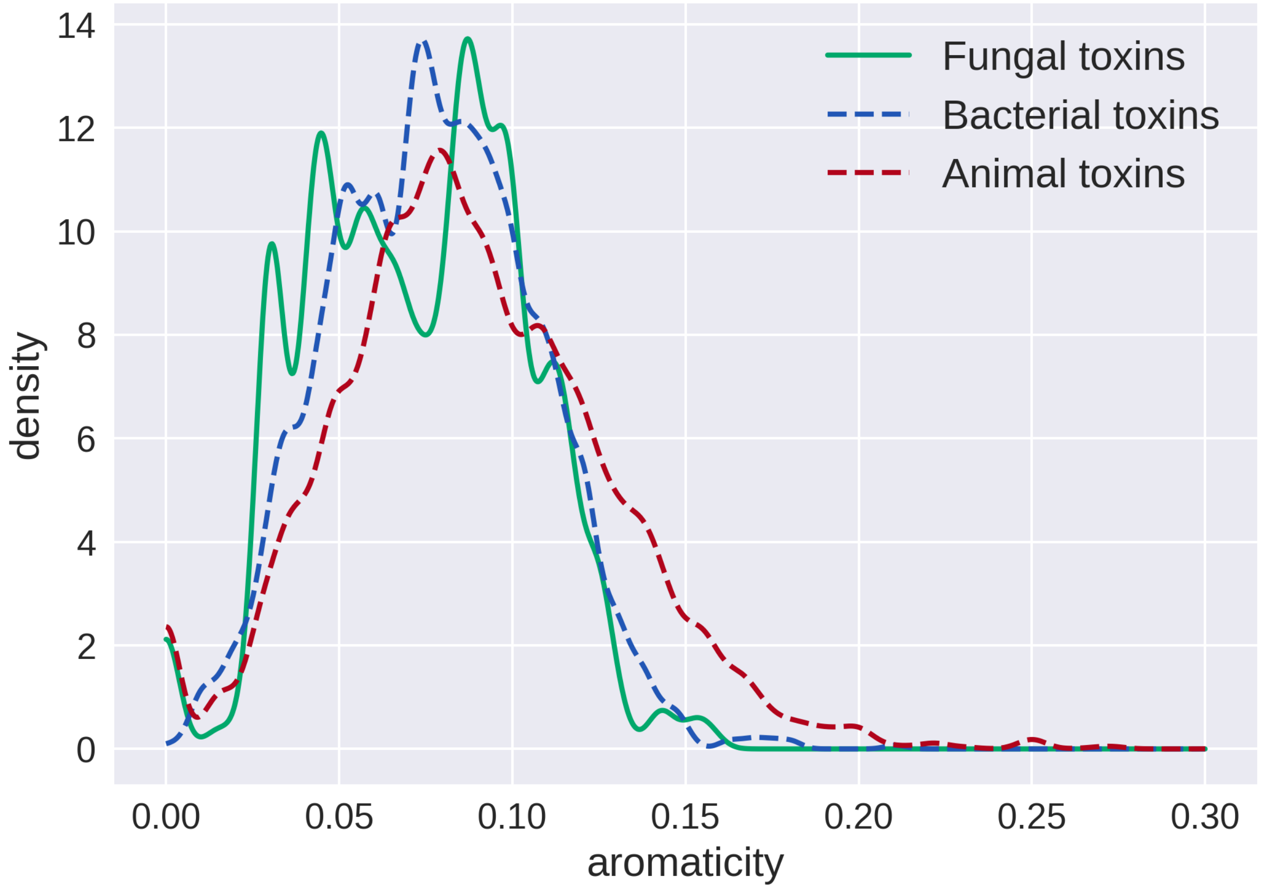

Fig. S8 : All toxins reveal similar aromaticity.

Comparison of the average aromaticity of proteins. Here aromaticity is the relative frequency of phenylalanine, tryptophan and tyrosine. Fungal toxins in green, bacterial toxins in blue and animal toxins in red.

Supplementary Method: Construction of the Fungal Toxin Dataset

The fungal toxin dataset was constructed using three database searches involving UniProt/Swiss-Prot (RRID:SCR_021164) and PubMed (RRID:SCR_004846), accessed in January 2025. The UniProt database was queried using the term “fungal peptide toxin.” Any hits corresponding to proteins involved in toxin synthesis, preprocessing, or members of toxin gene clusters without a direct toxic effect were manually excluded. Additionally, any erroneous hits that did not originate from fungi were removed.

A complementary search was performed using the intersection of the terms [keyword:KW-0800] (toxin) and [taxonomy id:4751] (fungi). For both sets, only reviewed entries were included in the final dataset. Proteins labeled as fragments or partial sequences were removed to ensure accurate length analyses.

The NCBI PubMed database was searched for entries with fungal origin and the term "toxin" in the title. Entries containing keywords such as "resistance," "resistant," "pump," "extrusion," "insensitivity," or "biosynthesis" were excluded. Additionally, putative, partial, and protein fragments were excluded. Subunits were also excluded due to missing annotation detail to which subunit contains the active part. Only entries from RefSeq were included for greater reliability. Proteins containing only non-toxic domains or mechanisms related to toxin insensitivity were also removed by manual curation.

The datasets from all three searches were combined. Since UniProt and NCBI databases can list proteins with identical amino acid sequences under different IDs, a redundancy reduction was performed using MMseqs2 with a threshold of 100% sequence similarity to remove duplicate entries.

For detailed information on the methods used for redundancy reduction and data analysis, refer to the "Data Set Comparison" section in the main article.
